# Vitamin B12 supplementation and health behavior of Austrian vegans: a cross-sectional online survey

**DOI:** 10.1038/s41598-023-30843-1

**Published:** 2023-03-22

**Authors:** Michelle Fuschlberger, Peter Putz

**Affiliations:** FH Campus Wien University of Applied Sciences, Favoritenstrasse 226, 1100 Vienna, Austria

**Keywords:** Nutrition, Public health

## Abstract

The number of vegans is increasing and was estimated at 2.0% of the Austrian population. Austrian vegans were found to have lower intakes and levels of vitamin B12 compared to vegetarians and omnivores. Vegans are advised to consume reliable sources of vitamin B12, e.g., in the form of dietary supplements or fortified foods. This study aimed to investigate health and supplementation behavior, with special emphasis on the supplementation of vitamin B12, and to demographically characterize the community of Austrian adult vegans. A nonrandom, voluntary sample of adult vegans with a principal residence in Austria was recruited with an online cross-sectional survey via social media and messenger platforms. Associations between respondent characteristics (gender, education, nutritional advice by a dietitian or nutritionist) and health/supplementation behaviors were examined by cross-tabulation. The questionnaire was completed by 1565 vegans (completion rate 88%), of whom 86% were female, the median age was 29 years, 6% were obese, and 49% had completed an academic education. Ninety-two percent consumed vitamin B12 through supplements and/or fortified foods, and 76% had their vitamin B12 status checked. The prevalence of vitamin B12 intake through supplements and/or fortified foods was slightly (not statistically significant) higher among women vs. men (93% vs. 89%), those who were academically educated vs. those who were not (93% vs. 91%), and those who had taken nutritional advice vs. those who had not (97% vs. 92%). Professional nutritional advice had been taken by only 9.5% of female and 8.4% of male respondents. Those who had taken advice reported a lower smoking prevalence (p = 0.05, φ = 0.05), higher prevalence of checking vitamin B12 status (p < 0.01, φ = 0.10), vit B12 intake through supplements and/or fortified foods (p = 0.03, φ = 0.05), and taking supplements of omega-3 (p < 0.01, φ = 0.14), selenium (p = 0.02, φ = 0.06), and iodine (p = 0.02, φ = 0.06). Austrian vegans can be characterized as predominantly young, female, urban, highly educated, and nonobese. The rate of vitamin B12 intake through supplements and/or fortified foods is fairly high (92%), but should be further improved e.g., by increasing the share of vegans who follow professional nutritional advice (requiring a diploma in dietetics, nutritional science, or medicine in Austria).

## Introduction

The number of vegans is increasing and was estimated to be 2.0% of the Austrian population in 2021^1^, 1.6% in Germany in 2016^2^, and 3.0% in the U.S. in 2020^3^; these estimates were based on varying survey dates and assessment methodologies and are therefore not directly comparable. An Austrian survey conducted in 2016 found a higher share of women among later adopters (past 3 years) than among earlier adopters (83% and 65%, respectively) and that the average age at vegan diet adoption was approximately 25 years^4^. For 86% of Austrian vegans, ethical motives (e.g., regarding factory farming) are the main consideration for choosing a vegan diet, ahead of health-related motives^5^. The FAO’s food balance sheets show a substantial decline in the Austrian pig meat supply (− 31%) and a slight decline in the bovine meat (− 8%) and poultry (− 5%) supply from 2010 to 2019^6^. It is the position of the US *Academy of Nutrition and Dietetics* that *“appropriately planned vegetarian, including vegan, diets are healthful, nutritionally adequate, and may provide health benefits in the prevention and treatment of certain diseases. These diets are appropriate for all stages of the life cycle, including pregnancy, lactation, infancy, childhood, adolescence, older adulthood, and for athletes …”*^7^*.* In contrast, the *German Nutrition Society* does not recommend a vegan diet for pregnant or breastfeeding women, babies, children, or adolescents in its position paper, due to uncertainty in research data^8,9^. However, the *German Society for Paediatric and Adolescent Medicine* stated that the nutritional needs of growing children and adolescents can generally be met through a balanced, vegetable-based diet^10^. Likewise, the Spanish Association of Paediatrics advises infants and young children to follow an omnivorous diet or, at least, an ovo-lacto-vegetarian diet^11^. Vegans may have lower intakes of vitamin B12 (henceforth “B12”), calcium, iodine, vit D, zinc, and long-chain omega-3 fatty acids^12–14^. Austrian vegans, in particular, were found to have lower intakes and levels of B12, vit B2, and calcium when compared to vegetarians and omnivores^5^. B12 status assessment and intake of reliable B12 sources, such as fortified foods or supplements, are recommended consistently to all vegans^7,8,15^. The *EFSA Panel on Dietetic Products, Nutrition and Allergies* (NDA) set an adequate intake for B12 at 4 μg/day for adults; for pregnant and lactating women, adequate intakes of 4.5 and 5 μg/day, respectively, were proposed^16^. If pregnant and/or lactating vegan women do not habitually use reliable B12 sources, they also place their offspring at risk for B12 deficiency^17^. B12 (cobalamin) is synthesized by certain bacteria and archaea but not by plants or animals. The synthesized B12 is transferred to and accumulates in animal tissues and even certain plant tissues (e.g. algae) through microbial interactions^18^. B12 is an essential molecule for humans, with functions in fatty acid, amino acid, and nucleic acid metabolic pathways; hypovitaminosis arises from inadequate absorption, genetic defects, or inadequate dietary intake^19^. B12 deficiency can present with nonspecific clinical features and in severe cases with neurological or hematological abnormalities^20^. However, it is important to emphasize that food supplements should only be used in cases in which supplementation is recommended (e.g. B12 supplementation recommended for vegans) and not as a general substitute for a healthy diet; it is advisable to consult a healthcare professional (e.g., a dietitian) and to make sure not to exceed the recommended dosage^21^. Users should be aware, that supplements can cause potential harm such as adverse reactions, drug interactions, monetary cost, delay of more effective therapy, false hope, and an increased medication burden^22^.

To date, there is a lack of knowledge about the health and supplementation behavior of Austrian vegans. This study aimed to investigate health and supplementation behavior, with special emphasis on the supplementation of B12, and to demographically characterize the community of Austrian adult vegans.

## Methods

### Respondents and survey administration

A nonrandom, voluntary sample of adult vegans with a principal residence in Austria was recruited with an online cross-sectional survey. The eligibility criteria for participation in the survey were (1) principal residence in Austria, (2) a current vegan diet, (3) age over 18 years, and (4) willingness to voluntarily participate in the survey. These criteria were queried at the beginning of the survey, and respondents who clicked the survey link but were not eligible were taken directly to the end of the survey. The online survey was created with the EFS Survey version 21.2 software (Tivian XI GmbH, Cologne, Germany). The online survey link was provided to Austrian vegan community groups, forums, and accounts via the social media and messenger platforms Instagram, Facebook, and WhatsApp. No compensation or incentives were offered for participation.

The survey included a total of 23 questions. The question regarding age (years) was asked in an open-ended response format, and gender was operationalized according to recommendations to address diversity^23^. Body mass index (BMI) was calculated (kg/m^2^) from self-reported body weight (kg) and height (cm). Respondents with a BMI of at least 30 were classified as obese. The International Standard Classification of Education (ISCED) 2011 was used for the operationalization of educational levels, and respondents with an ISCED level of at least 6 (i.e., bachelor’s degree) were classified as “academically educated”. Self-rated general health was queried as in the Austrian wave of the European Health Interview Survey^24^. The survey tracked the proportion of respondents who currently resided in the capital Vienna. With a population of approximately 1.9 million, Vienna is by far the country's largest city, while the next largest cities have fewer than 300,000 inhabitants. Items on supplementation behavior were used as in an online survey on the use of supplements and fortified foods among German vegans conducted in 2016^2^. The outcomes “B12 supplementation”, “vit B complex supplementation”, “multivitamin supplementation”, and “B12 fortified foods” were combined into an outcome expressing whether any B12 was consumed through supplements and/or fortified foods. To our knowledge, these items have not been tested for reliability or validity. Our survey, before being applied in this study, underwent face validity pretesting in a sample of ten individuals who provided feedback on the understandability, functionality, content, and completion time. Participation in the survey was anonymous and voluntary and could be terminated at any time without justification. The questionnaire was online for a period of 2 months from January until March 2022. There was no randomization or alternation of items, as later questionnaire sections built on responses and information given in the earlier sections. All items included a nonresponse option “don’t know/prefer not to say”. Respondents had the option to save and continue later, as well as to change their answers using the "Back" button. Duplicate participation by the same respondent was avoided by the use of cookies. Responses were stored in the online survey system after the last page of the survey was completed. At the time of conducting this research, anonymous surveys did not require formal review by a research ethics committee under Austrian research governance, in which the Declaration of Helsinki defines applicability to research on identifiable human data^25^. The survey followed ethical research practices (i.e., voluntary participation; reassurance of anonymity, data protection, and confidentiality; advance information on purpose and content; provision of contact details of the research team; and full disclosure of involved organizations). This information was summarized on the first page of the online survey. Anonymous electronic consent to voluntary participate was required to begin the survey, but no signatures were obtained. Reporting follows the Consensus-Based Checklist for Reporting of Survey Studies (CROSS)^26^.

### Statistical analysis

Responses were exported to SPSS statistical software version 27 (IBM Corp., 2021 Armonk, NY). Kolmogorov‒Smirnov tests and examination of quantile‒quantile plots were performed to assess the normality of metric data. Descriptive data are presented as medians with interquartile ranges (due to the violated normality assumption) or as frequencies with percentages. Subgroup analysis examined associations between respondent characteristics and health/supplementation behaviors by cross-tabulation. Twenty-one outcome items on health and supplementation behavior (as shown in Tables 1, 2, 3) were thereby compared in three dichotomous subgroup analyses by gender (male/female), completed academic education (yes/no), and ever taken professional nutritional advice by a dietitian or nutritionist (yes/no), giving a total of 63 statistical tests. Otherwise, no adjustments for confounders or sensitivity analyses were applied. These association analyses were prespecified but not based on a prospective sample size calculation. Because of the online survey design, a larger sample size did not cause any additional effort or cost. Thus, a sample size that was as large as possible was aimed for. A larger sample size implies that smaller effects reach statistical significance. The observed effects were therefore interpreted carefully with a focus on their effect sizes (comparison of percentages and φ). Due to a lack of knowledge about the distribution of participant characteristics in the vegan population, no adjustments for nonrepresentative samples, such as weighting of items, were made in the analysis. Exact chi-squared-based p values were reported, rounded to three decimal places. The multiplicity of testing (63 tests applied to the sample, with alpha 0.05) was corrected by the Bonferroni method, with a p value < 0.0008 consequently indicating statistical significance.

**Table 1:** Respondent characteristics.

	Female (n = 1349)	Male (n = 204)	Divers (n = 4)	Overall (n = 1565)
Age, MDN (IQR)	29.0 (11.0)	31.0 (10.0)	27.0 (9.0)	29.0 (12.0)
BMI, MDN (IQR)	21.9 (4.2)	23.3 (3.9)	22.1 (11.5)	22.2 (4.5)
Obese, %	5.9	7.8	25.0	6.3
Academically educated, %	49.3	44.8	50.0	48.6
Vegan + 1 year, %	87.7	90.7	100.0	88.2
Took advice, %	9.5	8.4	0.0	9.4
From Vienna, %	66.0%	67.3%	66.7%	66.3%

BMI (body mass index) based on self-reported indications of body weight and size and calculated as kg/m2.

“obese” classified by a body mass index greater than 30 kg/m^2^.

N = 1565, except for the following items, where “don’t know/wish not to say”, was selected 8 times for “gender”, 11 times for “academically educated”, 3 times for “vegan since + 1 year”, 4 times for “took advice”, and 380 times for “participating from Vienna”.

*MDN* median, *IQR* interquartile range.

## Results

### Respondents

Approximately 2% of Austrian adults (i.e., approximately 145,000 individuals) follow a vegan diet^1^ and were therefore potentially eligible to participate. The link to the online survey was accessed by 2373 individuals, of whom 2027 began the survey after reading the introductory information. After verifying eligibility based on questions about age, vegan diet, and principal residence in Austria, 1784 individuals remained in the sample. Of these 1784 individuals, 1565 completed a valid questionnaire, resulting in a completion rate of 87.7%. Invalid data points include missing and implausible values (e.g., body weight of 851 kg, height of 58 cm), whereas the “don’t know/prefer not to say” selections were processed and reported in the analysis.

Survey respondents were fairly young (median 29 years), although up to 74 years of age and predominantly female (86%), and current residence in the federal capital Vienna was strongly overrepresented. Nearly half of the respondents (49%) had completed an academic education at a bachelor’s level or higher, compared to 13% in the general adult population^27^. Only 6% of the sample was obese, compared to 17% in the general adult population^28^. BMI was below 18.5 kg/m^2^ among 101 (7%) of the respondents. Although recommended for vegans by the German Nutrition Society^9^, only 9% of the respondents had already taken professional nutritional advice. In summary, Austrian vegans can be characterized as predominantly young, female, urban, highly educated, and nonobese (Table 1).

### Health and supplementation behavior

General health status was self-rated as “very good” or “good” by 90.0% of the respondents, and 76.7% self-rated their dietary behavior as “healthy” or “somewhat healthy”. Regular participation in sports (i.e., at least three times a week) was reported by 62.2% of respondents. Regular alcohol consumption (i.e., at least several times a week) was reported by 6.4%, and 12.9% reported daily smoking. B12 status was checked at least once a year by 75.6% of respondents. Sixteen percent said they did not undergo any micronutrient status assessment, and another 22.2% did so less frequently than once a year. B12 supplements were taken by 82.2%, and 92.1% had any B12 intake through supplements and/or fortified foods. Other supplements that respondents took were: vit D (79.6%), omega-3 (50.8%), iron (49.5%), zinc (41.3%), protein (36.4%), calcium (35.8%), selenium (32.4%), vit B complex (31.7%), multivitamin (31.6%), iodine (29.1%), vit B2 (24.7%), and others (15.7%). Eight percent reported never taking nutritional supplements. Thus, the prevalence of supplement use was generally high and higher for individual components than for combined products.

The prevalence of “very good” or “good” general health was fairly similar among male (88.2%) and female (90.5%) respondents. This prevalence was lower in the general adult population (75%) but correspondingly similar across genders^24^. The prevalence of regular drinking (at least several times a week) was higher among male vegans than among female vegans (10.8% and 5.8%, respectively, p = 0.009) but markedly lower than in the general adult population (52% and 28%, respectively)^24^. The prevalence of daily smoking was similar among male (12.9%) and female (13.1%) respondents. In contrast, in the general adult population, this prevalence is higher among men (24 ß%) than among women (18%)^24^. Statistically significant differences in health and supplementation behaviors between genders were found for healthy eating behavior, regular drinking, checking B12 status, B12 supplementation, and iron supplementation with women showing more favorable behavior for all parameters; notably, women of childbearing age have an elevated requirement for iron^29^. The use of other dietary supplements was similar for both genders (Table 2). Due to a small number (n = 4) of respondents of diverse genders, the interpretability of their behaviors was limited. However, three respondents (75%) reported checking their B12 status, and four (100%) consumed B12 through supplements and/or fortified foods.

**Table 2:** Health and supplementation behavior, by gender (n = 1553).

	Overall^a^ (n)	%	Male (n)	%	Female (n)	%	|φ|	p
Good health	1408	90.0	180	88.2	1220	90.5	0.03	0.312
Healthy eating behavior	1196	76.6	138	67.6	1054	78.4	0.09	< 0.001**
Regular drinking	100	6.4	22	10.8	77	5.8	0.07	0.009*
Daily smoking	199	12.9	26	12.9	173	13.1	0.01	1.0
Regular sports	963	62.2	124	61.1	835	62.6	0.01	0.698
B12 status check	1176	75.5	136	66.7	1037	76.9	0.08	0.002*
B12 supplements	1287	82.2	155	76.0	1124	83.3	0.07	0.011*
B12 supplements/fortified foods	1442	92.1	182	89.2	1240	92.6	0.04	0.123
D supplements	1245	79.6	154	75.5	1081	80.1	0.04	0.136
Omega-3 supplements	795	50.8	101	49.5	687	50.9	0.01	0.708
Iron supplements	775	49.5	75	36.8	695	51.5	0.10	< 0.001**
Zinc supplements	646	41.3	84	41.2	559	41.4	0.00	1.0
Protein supplements	570	36.4	85	41.7	480	35.6	0.04	0.101
Calcium supplements	561	35.8	76	37.3	481	35.7	0.01	0.659
Selenium supplements	507	32.4	66	32.4	440	32.6	0.00	1.0
B complex supplements	496	31.7	61	29.9	432	32.0	0.02	0.573
Multivitamin supplements	494	31.6	74	36.3	417	30.9	0.04	0.126
Iodine supplements	455	29.1	59	28.9	391	29.0	0.00	1.0
B2 supplements	387	24.7	53	26.0	334	24.8	0.01	0.728
Other supplements	245	15.7	30	14.7	214	15.9	0.01	0.683
Not taking any supplements	128	8.2	21	10.3	107	7.9	0.03	0.273

Exact, Chi-squared based p values.

^a^Overall includes besides male and female participants also participants of diverse genders (n = 4) and without an indication in this regard (n = 8).

*Statistically significant at the alpha level of 0.05, ** statistically significant when accounting for the multiplicity of statistical testing by the Bonferroni method, accounting for 63 tests performed on the sample, resulting in p values < 0.0008 considered statistically significant.

All items self-rated, “good health” combines the options “very good” and “good”, “healthy eating behavior” combines the options “healthy” and “rather healthy”, “regular drinking” combines options at least as frequent as once times a week, “regular sports participation” combines options at least as frequent as three times a week, “B12 suppl. or fortified foods” combines intake of single B12 and/or complex vit B and/or multivitamin supplements and/or B12 fortified foods.

n = 1553, except for the following items, where “don’t know/wish not to say”, was selected once for “good health”, 4 times for “healthy eating behavior”, 14 times for regular drinking, 27 times for daily smoking, and 16 times for regular sports.

Academically educated respondents (i.e., ISCED levels 6 to 8), rated their eating, smoking, and sports behaviors significantly better than less educated respondents. The prevalence of B12 and vit D supplementation was slightly higher among academically educated respondents, whereas the use of other supplements was similar (Table 3).

**Table 3:** Health and supplementation behavior, by whether or not an academic education was completed (n = 1554).

	No academic education (n)	%	Academic education (n)	%	|φ|	p
Good health	715	89.6	682	90.3	0.01	0.673
Healthy eating behavior	581	73.2	608	80.4	0.09	< 0.001**
Regular drinking	47	6.0	52	6.9	0.02	0.468
Daily smoking	123	15.7	72	9.5	0.09	< 0.001**
Regular sports	465	58.9	493	65.9	0.07	0.005*
B12 status check	589	73.8	588	77.8	0.05	0.076
B12 supplements	637	79.8	642	84.9	0.07	0.009*
B12 supplements/fortified foods	728	91.2	704	93.1	0.04	0.187
D supplements	619	77.6	618	81.7	0.05	0.044*
Omega-3 supplements	405	50.8	384	50.8	0.00	1.0
Iron supplements	385	48.2	386	51.1	0.03	0.287
Zinc supplements	323	40.5	317	41.9	0.02	0.571
Protein supplements	287	36.0	278	36.8	0.01	0.752
Calcium supplements	277	34.7	281	37.2	0.03	0.315
Selenium supplements	246	30.8	257	34.0	0.03	0.193
B complex supplements	246	30.8	243	32.1	0.01	0.585
Multivitamin supplements	254	31.8	239	31.6	0.00	0.957
Iodine supplements	221	27.7	231	30.6	0.03	0.219
B2 supplements	194	24.3	188	24.9	0.01	0.814
Other supplements	121	15.2	123	16.3	0.02	0.577
Not taking any supplements	79	9.9	47	6.2	0.07	0.009*

Exact, Chi-squared based p values.

*Statistically significant at the alpha level of 0.05, **statistically significant when accounting for the multiplicity of statistical testing by the Bonferroni method, accounting for 63 tests performed on the sample, resulting in p values < 0.0008 considered statistically significant.

All items self-rated, “good health” combines the options “very good” and “good”, “healthy eating behavior” combines the options “healthy” and “rather healthy”, “regular drinking” combines options at least as frequent as once times a week, “regular sports participation” combines options at least as frequent as three times a week, “B12 suppl. or fortified foods” combines intake of single B12 and/or complex vit B and/or multivitamin supplements and/or B12 fortified foods.

n = 1554, except for the following items, where “don’t know/wish not to say”, was selected once for “good health”, 4 times for “healthy eating behavior”, 14 times for regular drinking, 27 times for daily smoking, and 16 times for regular sports.

Professional nutritional advice had been taken by only 9.5% of female and 8.4% of male respondents. Those who had received such advice reported a lower smoking prevalence, higher prevalence of checking B12 status, consuming B12 through supplements and/or foods as well as taking omega-3, selenium, and iodine supplements. Only 3.4% of those who took professional nutritional advice, did not consume B12 through supplements and/or fortified foods, and 11.6% did not have their B12 status checked (Table 4).

**Table 4:** Health and supplementation behavior, by whether or not ever taken professional nutritional advice (n = 1561).

	No nutritional advice (n)	%	Nutritional advice (n)	%	|φ|	p
Good health	1279	90.4	125	86.2	0.04	0.144
Healthy eating behavior	1072	76.0	120	82.2	0.04	0.101
Regular drinking	90	6.4	9	6.2	0.00	1.0
Daily smoking	188	13.5	11	7.6	0.05	0.050*
Regular sports	865	61.7	94	65.3	0.02	0.419
B12 status check	1050	74.2	129	88.4	0.10	< 0.001**
B12 supplements	1159	81.9	124	84.9	0.02	0.369
B12 supplements/fortified foods	1297	91.7	141	96.6	0.05	0.035*
D supplements	1116	78.9	125	85.6	0.05	0.066
Omega-3 supplements	688	48.6	105	71.9	0.14	< 0.001**
Iron supplements	704	49.8	68	46.6	0.02	0.487
Zinc supplements	578	40.8	67	45.9	0.03	0.252
Protein supplements	508	35.9	61	41.8	0.04	0.176
Calcium supplements	504	35.6	56	38.4	0.02	0.526
Selenium supplements	446	31.5	60	41.1	0.06	0.020*
B complex supplements	454	32.1	41	28.1	0.03	0.351
Multivitamin supplements	443	31.3	50	34.2	0.02	0.513
Iodine supplements	399	28.2	55	37.7	0.06	0.017*
B2 supplements	354	25.0	31	21.2	0.03	0.317
Other supplements	216	15.3	28	19.2	0.03	0.231
Not taking any supplements	120	8.5	8	5.5	0.03	0.266

Exact, Chi-squared based p values.

*Statistically significant at the alpha level of 0.05, ** statistically significant when accounting for the multiplicity of statistical testing by the Bonferroni method, accounting for 63 tests performed on the sample, resulting in p values < 0.0008 considered statistically significant.

All items self-rated, “good health” combines the options “very good” and “good”, “healthy eating behavior” combines the options “healthy” and “rather healthy”, “regular drinking” combines options at least as frequent as once times a week, “regular sports participation” combines options at least as frequent as three times a week, “B12 suppl./fortified foods” combines intake of single B12 and/or complex vit B and/or multivitamin supplements and/or B12 fortified foods.

n = 1561, except for the following items, where “don’t know/wish not to say”, was selected 5 times for “good health”, 4 times for “healthy eating behavior”, 14 times for regular drinking, 27 times for daily smoking, and 16 times for regular sports.

## Discussion

This study provides novel insights into the health and supplementation behaviors as well as demographic characteristics of Austrian adult vegans. As in our study, in which vegans were predominantly female and young, vegetarianism was found to be three times more common among young adults in the U.S. (18–34 years) than among people over 65 years of age^30^. The share of female vegans (86%) and academically educated vegans (49%) in our study was roughly similar to data from a recent Swiss survey: 83% female, and 54% academically educated^31^. Self-rated health status and health behavior were markedly better in vegans than among the general Austrian adult population. Within the population of Austrian vegans, beneficial health behaviors and adherence to recommended checkups and supplementation of B12 were more prevalent among those respondents who were female, academically educated, and those who had taken nutritional advice. In contrast, general health status was not associated with these respondent characteristics. The prevalence of supplement use was generally high and higher for individual components than for combined products. The most frequently taken supplements were B12 (82%), vit D (80%), omega-3 (51%), and iron (50%). B12 supplementation was similarly assessed for German vegans (82%)^2^ and Czech children (86%)^32^. In a survey of healthcare professionals who were physically present at an international conference on plant-based nutrition, 98% of vegans self-reported supplementing B12^33^. Within our sample, 92% consumed B12 through supplements and/or fortified foods, and 76% had their B12 status checked at least once a year.

### Limitations

This study recruited a nonrandom voluntary response sample via social media and messenger platforms, which introduces a risk of selection bias and limits the generalizability of the findings. The recruitment channels might have better reached younger recipients and those who frequently use social media. Since the distribution of demographic characteristics in the vegan population is not known, representativeness cannot be assessed by matching the sample to the population. Pregnancy or breastfeeding was not assessed or screened out. While most questions asked about usual behaviors, the questions on supplementation were on the current situation and might therefore have been biased by pregnancy or breastfeeding-related supplementation behaviors. Comparisons with other study results were limited by varying survey dates and methodologies.

### Implications for future research

To obtain comparable data on the supplementation behaviors of vegans, standardized cross-national assessments are needed. Some societies do not recommend vegan diets for pregnant or breastfeeding women, babies, children, and adolescents, due to uncertainty in research data. Existing evidence suggests that the health of people who follow plant-based diets appears to be generally good, with several advantages but also some risks (hemorrhagic stroke, bone fracture)^14^. The extent to which these risks can be mitigated in certain populations (particularly children and pregnant or lactating women) by optimal food choices, fortification, and supplementation remains subject to future research.

## Conclusions

Austrian vegans can be characterized as predominantly young, female, urban, highly educated, and nonobese. The rate of B12 intake through supplements and/or fortified foods is fairly high (92%) but should be further improved. Further improvement could be achieved by increasing the share of vegans who follow nutritional advice from health care professionals (requiring a diploma in dietetics, nutritional science, or medicine in Austria), and these professionals should put emphasis on recommending B12 intake through supplements and/or fortified foods and B12 status checks.

## Data Availability

The datasets used and/or analyzed during the current study are available from the corresponding author on reasonable request.

## Abbreviations

EFSA: European Food Safety Authority
FAO: Food and Agricultural Organization of the United Nations
IQR: Interquartile range
ISCED: International Standard Classification of Education
MDN: Median
NDA: EFSA Panel on Dietetic Products, Nutrition and Allergies
vit: Vitamin
U.S.: United States
